# Targeting hyperactivation of the AKT survival pathway to overcome therapy resistance of melanoma brain metastases

**DOI:** 10.1002/cam4.50

**Published:** 2013-02-03

**Authors:** Heike Niessner, Andrea Forschner, Bernhard Klumpp, Jürgen B. Honegger, Maria Witte, Antje Bornemann, Reinhard Dummer, Annemarie Adam, Jürgen Bauer, Ghazaleh Tabatabai, Keith Flaherty, Tobias Sinnberg, Daniela Beck, Ulrike Leiter, Cornelia Mauch, Alexander Roesch, Benjamin Weide, Thomas Eigentler, Dirk Schadendorf, Claus Garbe, Dagmar Kulms, Leticia Quintanilla-Martinez, Friedegund Meier

**Affiliations:** 1 Division of Dermatologic Oncology, Department of Dermatology, University of TuebingenGermany; 2 Department of Diagnostic and Interventional Radiology, University of TuebingenGermany; 3 Department of Neurosurgery, University of TuebingenGermany; 4 Department of Surgery, University of TuebingenGermany; 5 Department of Neuropathology, University of TuebingenGermany; 6 Department of Dermatology, University Hospital ZurichZurich, Switzerland; 7 Institute of Pathology, University of TuebingenGermany; 8 Department of Neurology, University Hospital ZurichZurich, Switzerland; 9 Massachusetts General Hospital Cancer CenterBoston, Massachusetts; 10 Department of Dermatology, University of CologneGermany; 11 Department of Dermatology, University of HomburgGermany; 12 Department of Dermatology, University of EssenGermany; 13 Department of Dermatology, University of DresdenGermany

**Keywords:** AKT, BRAF inhibitors, brain metastasis, melanoma, therapy resistance

## Abstract

Brain metastases are the most common cause of death in patients with metastatic melanoma, and the RAF-MEK-ERK and PI3K-AKT signaling pathways are key players in melanoma progression and drug resistance. The BRAF inhibitor vemurafenib significantly improved overall survival. However, brain metastases still limit the effectiveness of this therapy. In a series of patients, we observed that treatment with vemurafenib resulted in substantial regression of extracerebral metastases, but brain metastases developed. This study aimed to identify factors that contribute to treatment resistance in brain metastases. Matched brain and extracerebral metastases from melanoma patients had identical ERK, p-ERK, and AKT immunohistochemistry staining patterns, but there was hyperactivation of AKT (p-AKT) and loss of PTEN expression in the brain metastases. Mutation analysis revealed no differences in BRAF, NRAS, or KIT mutation status in matched brain and extracerebral metastases. In contrast, AKT, p-AKT, and PTEN expression was identical in monolayer cultures derived from melanoma brain and extracerebral metastases. Furthermore, melanoma cells stimulated by astrocyte-conditioned medium showed higher AKT activation and invasiveness than melanoma cells stimulated by fibroblast-conditioned medium. Inhibition of PI3K-AKT signaling resensitized melanoma cells isolated from a vemurafenib-resistant brain metastasis to vemurafenib. Brain-derived factors appear to induce hyperactivation of the AKT survival pathway and to promote the survival and drug resistance of melanoma cells in the brain. Thus, inhibition of PI3K-AKT signaling shows potential for enhancing and/or prolonging the antitumor effect of BRAF inhibitors or other anticancer agents in melanoma brain metastases.

## Introduction

The prognosis for melanoma patients with distant metastases is poor, with a median overall survival time of about 8 months [1], reflecting the failure of the chemotherapy and immunotherapy regimens that were used in the past. However, basic research has shown that the RAF-MEK-ERK and PI3K-AKT signaling pathways are key players in melanoma progression and drug resistance [2,3]. A recent phase III study showed that the BRAFV600E kinase inhibitor vemurafenib induced partial or complete tumor regression in 48% of patients with BRAFV600E-mutated metastatic melanoma as compared with 5% of patients treated with the classical chemotherapeutic agent dacarbazine [4]. Moreover, in a phase II trial of vemurafenib with a long follow-up, the median overall survival was approximately 16 months [5]. However, brain metastases still limit the effectiveness of this therapy.

Brain metastases occur in over 70% of patients with metastatic melanoma and are the most common cause of death. The overall survival of melanoma patients with brain metastases is generally very poor, with a median survival time of 5 months [6]. Current therapeutic options include neurosurgery, radiosurgery, whole-brain radiation, and chemotherapy. Patients treated with neuro- or radiosurgery appear to have a longer median survival of about 9 months [7,6]. Chemotherapeutic agents such as temozolomide that are used for treating primary brain tumors are not effective for cerebral melanoma metastases [8]. Intriguingly, a recent phase 2 trial showed that immunotherapy with ipilimumab has some activity in melanoma brain metastases, particularly when brain metastases are small and asymptomatic [9]. Furthermore, recent and ongoing clinical trials show clinical activity of BRAF inhibitors in patients with asymptomatic melanoma brain metastases [10,11]. However, effects of BRAF inhibitors in melanoma brain metastases appear to be limited.

We observed in a series of patients that treatment with the BRAF inhibitor vemurafenib yielded a substantial response in extracerebral metastases, but brain metastases developed. The aim of this study was thus to identify factors that contribute to the relative treatment resistance of brain metastases.

## Materials and Methods

### Isolation and culture of human cells

The use of human tissue was approved by the medical ethics committee of the University of Tuebingen (Project Number 017/2012BO2) and was performed in accordance with the Principles of the Declaration of Helsinki. Cell lines and melanoma cells from excised brain or extracerebral metastases were isolated and cultured as described previously [12–14]. Human fibroblasts were isolated from human foreskin and cultured in Dulbecco's modified Eagle's medium (DMEM) with 10% fetal bovine serum (FBS) [14]. Immortalized human fetal astrocytes (SV-FHA) were kindly provided by Dr. Muruganandam (Institute of Biological Sciences, National Research Council of Canada) and were cultured in DMEM with FBS.

### Stimulation with conditioned medium

To obtain conditioned medium, fibroblasts and astrocytes were grown for 24 h in Roswell Park Memorial Insitute (RPMI) without serum. The medium was collected and frozen at −80°C until use. Prior to stimulation, melanoma cells were seeded at a density of 250,000 cells per well in a six-well plate. The next day, conditioned media from fibroblasts and astrocytes were added; RPMI without serum was added to control cells. The cells were harvested and lysed after 1, 3, and 6 h of stimulation.

### Signaling pathway inhibitors

Signaling pathway inhibitors included the BRAFV600E kinase inhibitor, vemurafenib (Selleck), and the PI3K inhibitor, GDC-0941 (Selleck, ICS International Clinical Service GmbH, MüFCnchen, Deutschland). Inhibitors were dissolved in dimethylsulfoxide, and stored at −20°C. Controls were incubated with culture medium or culture medium and DMSO (Dimethylsulfoxid).

### Immunohistochemistry

For immunohistochemistry, human tumor tissue was fixed in 4% formalin and embedded in paraffin. The following primary antibodies were used: anti-ERK, anti-phospho-ERK (Thr202/Tyr204), anti-AKT, anti-phospho-AKT (Thr308), anti-PTEN, anti-Melan-A (Cell Signaling Technology Inc., Beverley, MA), anti-CD14 (Cellmark, Medac, Gothenburg, Sweden), anti-GFAP (Clone6F2), and anti-HMB45 (DAKO, Hamburg, Germany). Specifically, Melan-A, glial fibrillary acidic protein (GFAP), HMB45, and CD14 were detected using the UltraView Universal DAB Detection Kit, and the other antibodies were detected using the UltraView Universal Alkaline Phosphatase Red Detection Kit from Ventana (Tucson, AZ). Quantification and scoring of blinded samples were done using LQ (Pathology), AB (Neuropathology), and FM (Dermatopathology).

### Matrigel cell invasion assay

Inserts (Corning Life Sciences, Amsterdam, the Netherlands) were coated with Matrigel (BD Biosciences, Heidelberg, Germany), and ZüMel1 and ZüMel1H melanoma cells were seeded onto the Matrigel matrix and fed with DMEM without FBS. The lower chamber of the transwell was filled with serum-free culture medium (ctrl), fibroblast- or astrocyte-conditioned medium (cm). After 24 h, the membrane was fixed and stained with hematoxylin. The cells that had migrated to the bottom surface of the membrane were counted.

### Western blot

Western blot analysis was performed as described previously [15]. The following primary antibodies were used: anti-ERK, anti-phospho-ERK (Thr202/Tyr204), anti-AKT, anti-phospho-AKT (Thr308), anti-PTEN, and anti-Actin (Cell Signaling, Beverley, MA). For quantification, Scion Image was used to compare phospho-AKT/Actin from the sample to phospho-AKT/Actin from the control. Cells newly isolated from brain and extracerebral distant metastases of melanoma patients were subjected to Western blot analysis within 14 days.

### Mutation analysis

For polymerase chain reaction (PCR) and sequencing, DNA was isolated from patient samples by proteinase K digestion. Subsequently, DNA was amplified with PCR using different primer sets (Table S2). Direct DNA sequencing was performed using the purified PCR products.

### Growth assay

Growth assays were performed as described previously [12,15].

### Apoptosis assay

The apoptosis assays were performed as described previously [12,15]. Each histogram represents at least 10,000 individual events (measured cells).

## Results

### Clinical observations

Our repeated observation is that patients with metastatic melanoma who are treated systemically with BRAF inhibitors show remission of extracerebral metastases, but development or progression of brain metastases. Here, we describe the clinical courses of two patients who received vemurafenib. Both patients had a complete remission of extracerebral metastases, but also showed development of new cerebral metastases.

#### Case report 1

**Figure 1:**
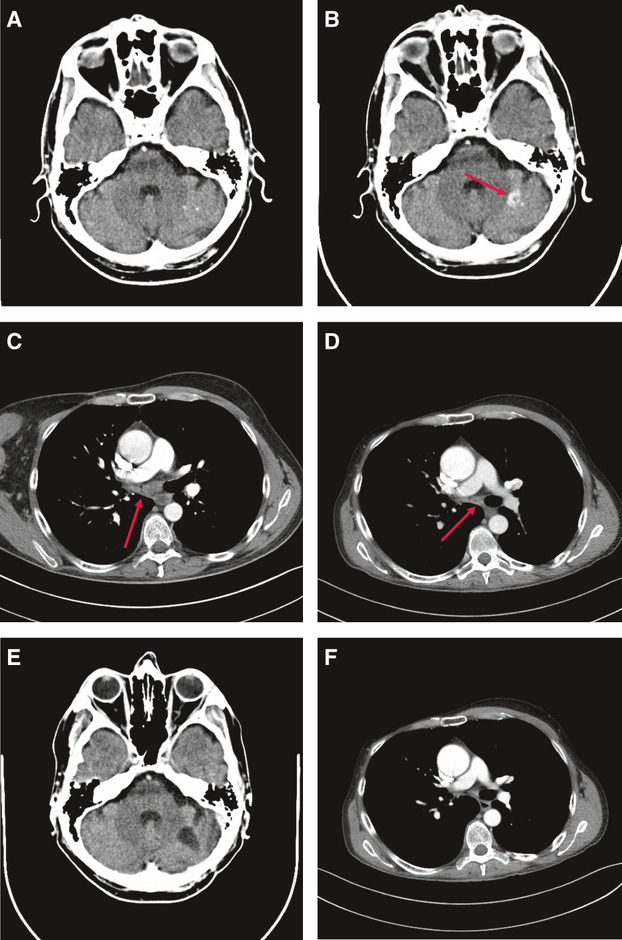
Computed tomography (CT) scans before and during treatment with vemurafenib (patient 1). (A) CT scan (August 2010) before treatment: no cerebral metastases. (B) CT scan (May 2011) during treatment: new cerebral metastasis. (C) CT scan (August 2010) before treatment: hilar lymph node metastases. (D) CT scan (May 2011) during treatment: regression. (E) CT scan (August 2012) during treatment: complete remission of extracerebral metastases. (F) CT scan (August 2012) during treatment: no cerebral metastases.

In August 2010, a 49-year-old man with BRAFV600E-mutated metastatic melanoma was enrolled into the BRIM-3 trial receiving the BRAFV600E kinase inhibitor vemurafenib. Computed tomography (CT) scans demonstrated a partial remission of the axillary, mediastinal, hilar, and lung metastases without evidence of brain metastases. However, a CT scan of the brain on May 2011 revealed a cerebellar metastasis on the left side (Fig. 1A and B) despite further regression of lymph node and lung metastases (Fig. 1C and D). The cerebellar metastasis was surgically removed, and therapy with vemurafenib was continued. The last CT imaging (28 August 2012) showed complete remission (Fig. 1E and F) (Table S1: Patient 1).

#### Case report 2

**Figure 2:**
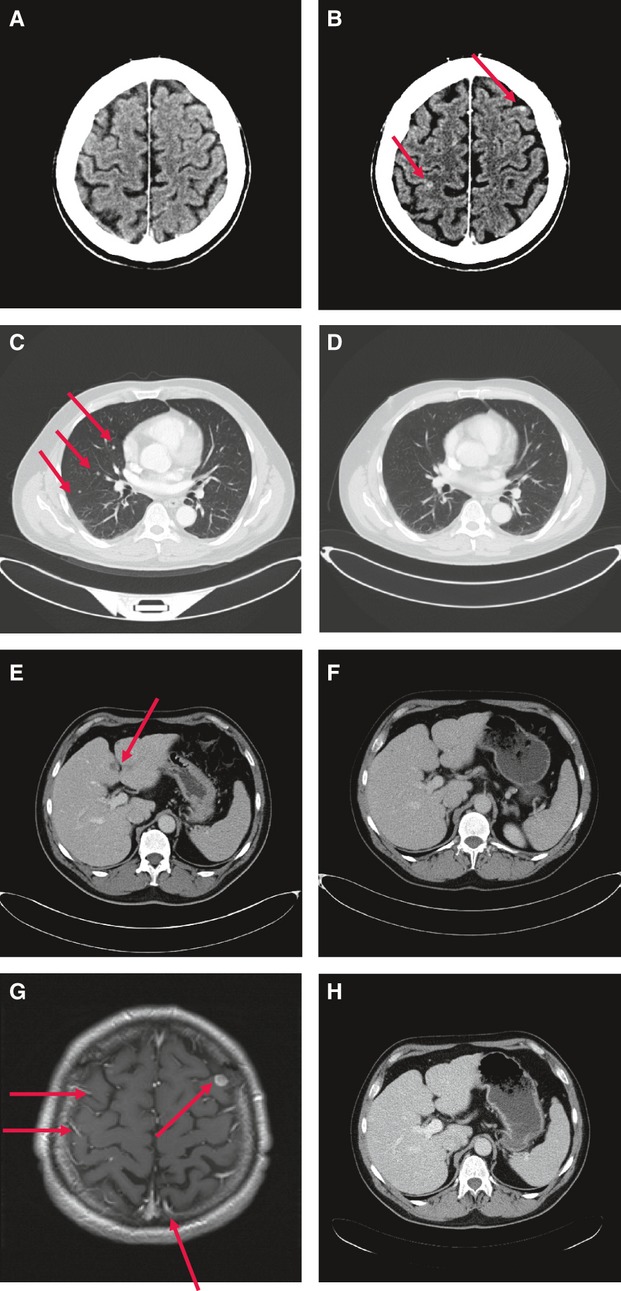
Computed tomography (CT) scans before and after treatment with vemurafenib (A–F) or ipilimumab (G and H) (patient 2). (A) CT scan (June 2010) before treatment: no cerebral metastases. (B) CT scan (August 2011) after treatment: new cerebral metastases. (C) CT scan (June 2010) before treatment: pulmonary metastases. (D) CT scan (August 2011) after treatment: complete remission. (E) CT scan (June 2010) before treatment: liver metastases. (F) CT scan (August 2011) after treatment: complete remission. (G) Magnetic resonance tomography (November 2011) after treatment with ipilimumab: progression of brain metastases with leptomeningeal metastases. (H) CT scan (November 2011) after treatment with ipilimumab: complete remission of liver metastases.

In June 2010, a 46-year-old man with BRAFV600E-mutated metastatic melanoma was recruited into the BRIM-3 trial receiving vemurafenib. After 4 months, the CT imaging showed a complete response of lung, liver, and lymph node metastases. In August 2011, the patient developed two brain metastases (Fig. 2A and B), although the extracerebral metastases of the patient were in complete remission (Fig. 2C–F). Vemurafenib was discontinued, and therapy with ipilimumab (3 mg/kg body weight) was started. Before the third cycle of ipilimumab treatment, cerebral and leptomeningeal metastases were diagnosed (Fig. 2G). Otherwise the other metastases of the patient were in complete remission (Fig. 2H). Three months later, the patient died as a result of brain metastases (Table S1: Patient 2).

Five other patients being treated with vemurafenib for metastatic melanoma developed new cerebral metastases, whereas the extracerebral metastases continued to respond to therapy (Table S1).

### Histological and mutational analysis

#### AKT is predominantly activated in melanoma brain metastases

**Figure 3:**
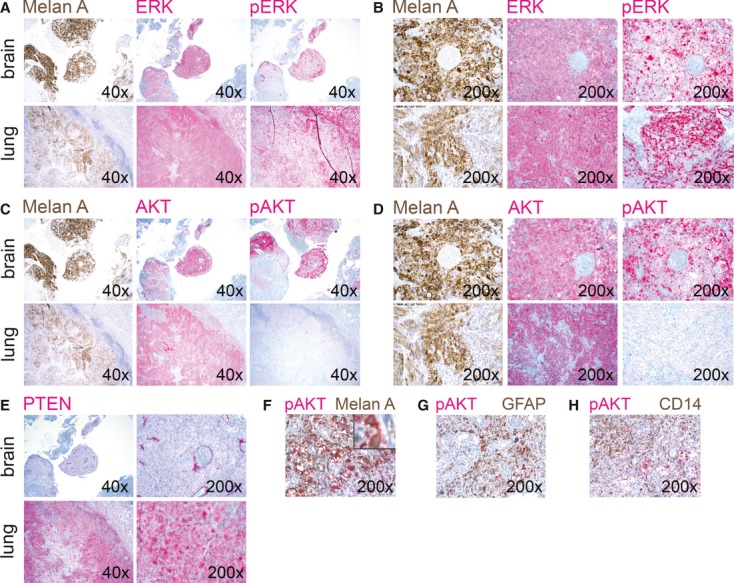
(A–E) Brain and lung metastases from a melanoma patient were stained to detect the melanocytic marker Melan A, total and activated ERK (p-ERK), total and activated AKT (p-AKT), and PTEN; 40×: 40-fold magnification, 200×: 200-fold magnification. (F) Brain metastasis from a melanoma patient was stained for p-AKT (red) and HMB-45 (brown) to detect melanoma cells, 200×: 200-fold magnification. The insert shows colocalization of p-AKT (red) with HMB-45 (brown), 630-fold magnification. (G) Brain metastasis from a melanoma patient was stained for p-AKT (red) and glial fibrillary acidic protein (GFAP) (brown) to detect glial cells, 200×: 200-fold magnification. GFAP does not colocalize with p-AKT. (H) Brain metastasis from a melanoma patient was stained for p-AKT (red) and CD14 (brown) to detect monocytes/macrophages, 200×: 200-fold magnification. CD14 does not colocalize with p-AKT.

As the RAF-MEK-ERK and PI3K-AKT signaling pathways are thought to be key players in melanoma progression and drug resistance, we performed immunohistochemical analyses of cerebral metastases and of matched extracerebral metastases from the same patients. All metastases were synchronously collected from patients with stage IV melanoma before initiating systemic treatment. The melanocyte marker Melan-A and the signal transduction molecules ERK, activated ERK (p-ERK), AKT, and activated AKT (p-AKT), and a major negative regulator of the PI3K-AKT pathway, the phosphatase and tensin homolog deleted from chromosome 10 (PTEN), were analyzed. Immunohistochemical analysis (Fig. 3A–D) showed that most cerebral and extracerebral metastases were positive for ERK and AKT throughout the entire tumor, while p-ERK was seen predominantly at the tumor periphery. Interestingly, most cerebral metastases were highly positive for activated AKT (p-AKT), whereas extracerebral metastases, in particular lung and liver metastases, present at the same time in the same patients were weakly positive or negative for activated AKT (Fig. 3C and D; Table 1). Moreover, the negative regulator of the PI3K-AKT pathway, PTEN, was downregulated in most brain metastases (Fig. 3E; Table 1). To determine where the p-AKT signal comes from, two-color immunohistochemical staining was performed. Figure 3F shows colocalization of p-AKT with the melanocytic marker HMB-45, indicating that in melanoma cells AKT is activated. Furthermore, we performed double immunohistochemistry for p-AKT and GFAP, a marker for glial cells, and for p-AKT and CD14, a monocyte/macrophage marker. As shown in Figure 3G and H, p-AKT does not colocalize with GFAP or CD14. Altogether, the immunohistochemical findings indicate that AKT is strongly activated in melanoma cells that have metastasized to the brain.

**Table 1:** Expression and activation of ERK, AKT, and PTEN in cerebral and matched extracerebral melanoma metastases

Patient sample	Melanoma metastases	ERK	p-ERK	AKT	p-AKT	PTEN
1	Brain	+++	++	+++	+++	−
	Lung	+++	+	+++	−	++
2	Brain	+++	+	+++	++	−
	Lymph node	++	+	+	−	NA
3	Brain	++	+	+	+++	−
	Lymph node	++	+	++	−	+
4	Brain	+++	+	+++	++	−
	Lymph node	+++	++	+++	−	+
5	Brain	+++	++	++	−	+
	Skin	+++	++	+++	−	+
6	Brain	+++	++	+++	+++	+
	Lung	+++	+	+++	−	NA
7	Brain	NA	NA	NA	+++	NA
	Liver	+	−	−	−	NA
8	Brain	NA	NA	NA	+++	NA
	Intestine	+++	+	++	+	NA
9	Brain	+++	++	++	+++	−
	Intestine	++	+	+	+	−

−, <10% of the cells positive; +, 10–20% of cells positive; ++, 20–50% of cells positive; +++, >50% of cells positive; NA, not available.

#### BRAF, NRAS, and KIT mutation status is identical in brain and extracerebral melanoma metastases

Mutational analysis of BRAF, NRAS, and KIT was performed on the same tumors (Table 2), which were analyzed by immunohistochemistry. BRAF mutations were detected in five patients, NRAS mutations were detected in two patients, and wild-type BRAF and NRAS genes were found in one patient. There was no difference in the BRAF, NRAS, or KIT mutation status in matched cerebral and extracerebral melanoma metastases, with one exception. In that patient, we detected wild-type BRAF and NRAS in the brain metastasis and the BRAFV600E mutation and wild-type NRAS in the matched liver metastasis.

**Table 2:** Mutational analysis of cerebral and matched extracerebral melanoma metastases

Patient sample	Melanoma metastases	BRAF	NRAS	KIT
1	Brain	V600K	WT	WT
	Lung	V600K	WT	WT
2	Brain	V600E	WT	WT
	Lymph node	V600E	WT	WT
3	Brain	WT	Q61R	WT
	Lymph node	WT	Q61R	WT
4	Brain	V600E	WT	WT
	Lymph node	V600E	WT	WT
5	Brain	WT	Q61H	WT
	Skin	WT	Q61H	WT
6	Brain	V600K	WT	WT
	Lung	V600K	WT	WT
7	Brain	WT	WT	WT
	Liver	V600E	WT	WT
8	Brain	V600K	WT	WT
	Intestine	V600K	WT	WT
9	Brain	V600R	WT	WT
	Intestine	V600R	WT	WT

### In vitro studies

#### AKT is activated in monolayer cultures established from brain and extracerebral melanoma metastases

We also determined the expression of ERK, p-ERK, AKT, p-AKT, and PTEN in vitro in monolayer cultures of established matched cerebral and extracerebral metastatic melanoma cell lines (ZüMel1H, ZüMel1, ZüMel2H, ZüMel2). Furthermore, we determined ERK, p-ERK, AKT, p-AKT, and PTEN expression in melanoma cells newly isolated from brain and extracerebral distant metastases of melanoma patients (TüMel32H, TüMel32; TüMel19H, TüMel21H, TüMel25H; TüMel22, TüMel23, TüMel27, TüMel28, TüMel30). In contrast to the immunohistochemical findings in the tumor tissues described above, there was no difference in AKT activation or PTEN expression status in brain and extracerebral metastatic melanoma cells from the monolayer cultures (Table 3). AKT, p-AKT, and PTEN were clearly expressed in both the established and the newly isolated cerebral and matched extracerebral metastatic melanoma cell lines with one exception showing loss of PTEN in cells isolated from a melanoma brain metastasis (Table 3).

**Table 3:** Expression and activation of AKT and PTEN in cerebral and extracerebral metastatic melanoma cell lines by Western blot analysis

	AKT	p-AKT	PTEN
ZüMel1H	++	+	+
ZüMel1	++	+	+
ZüMel2H	++	++	+
ZüMel2	++	++	+
TüMel32H	++	+	+
TüMel32	++	+	+
TüMel19H	++	+	−
TüMel21H	++	+	++
TüMel25H	++	+	++
TüMel22	++	+	+
TüMel23	++	+	+
TüMel27	++	+	++
TüMel28	++	+	+
TüMel30	++	+	+

H, cerebral; +, expressed; ++, highly expressed.

#### Astrocyte-conditioned medium stimulates AKT activation and invasiveness in melanoma cells

**Figure 4:**
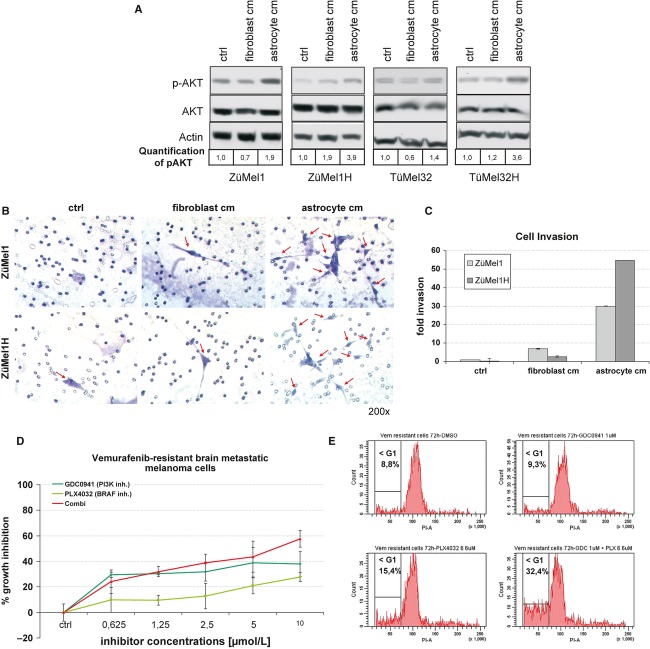
(A) Melanoma cells derived from patients with extracerebral metastases (ZüMel1, TüMel32) and matched brain metastases (ZüMel1H, TüMel32H) were treated with culture medium without serum as control (ctrl), fibroblast- or astrocyte-conditioned media (cm). The expression of total and activated AKT (p-AKT) and β-actin was analyzed by Western blot analysis. The bottom row shows quantification of p-AKT levels. One representative experiment is shown (three independent experiments). (B) Melanoma cells derived from a patient with extracerebral (ZüMel1) and matched brain (ZüMel1H) metastases were subjected to a transwell matrigel invasion assay. The lower chamber of the transwell was filled with serum-free culture medium (ctrl), fibroblast- or astrocyte-conditioned medium (cm). Arrows indicate melanoma cells that invaded through the Matrigel matrix. (C) Quantification of transwell matrigel invasion assay. The number of melanoma cells invaded through the Matrigel matrix is expressed as fold change compared with the control (ctrl, ZüMel1, serum-free medium). One representative experiment is shown (mean ± SD of duplicates, three independent experiments). (D) Growth assessment (4-methylumbelliferyl heptanoate) of vemurafenib-resistant brain metastatic melanoma cells treated with the indicated concentrations of the BRAF inhibitor vemurafenib or/and the PI3K inhibitor GDC0941 for 72 h. The percentage of growth inhibition was compared to DMSO-treated controls. One representative experiment is shown (mean ± SD, three independent experiments). (E) Vemurafenib-resistant brain metastatic melanoma cells were treated with the BRAF inhibitor vemurafenib or/and the PI3K inhibitor GDC0941, or DMSO (control) for 72 h. Apoptosis (<G1, subG1 fraction) was quantified by propidium iodide staining. One representative experiment is shown (three independent experiments).

To further investigate the differences in the findings in tumor tissues and tumor cell lines, cerebral and matched extracerebral metastatic melanoma cells were stimulated by two kinds of conditioned media. First, to simulate the tumor environment of brain metastases, melanoma cells were stimulated by astrocyte-conditioned medium. Second, to simulate the tumor environment of extracerebral metastases, cells were stimulated by fibroblast-conditioned medium. Melanoma cells grown in culture medium served as controls. Most interestingly, both cerebral and matched extracerebral metastatic melanoma cells stimulated by astrocyte-conditioned medium showed a higher activation of AKT (p-AKT) than cells stimulated by fibroblast-conditioned medium (Fig. 4A).

To evaluate the invasive competence of melanoma cells in response to stimulation by astrocyte-conditioned medium, a transwell matrigel invasion assay was employed. Astrocyte-conditioned medium strongly increased invasiveness of melanoma cells compared with fibroblast-conditioned medium or serum-free medium (Fig. 4B and C).

#### PI3K inhibition sensitizes melanoma cells isolated from a vemurafenib-resistant brain metastasis to vemurafenib

To study the effects of PI3K inhibition on vemurafenib-resistant brain metastatic melanoma cells, we isolated melanoma cells from a brain metastasis in a patient who was treated with vemurafenib and had a complete remission of extracerebral metastases, but developed a new brain metastasis. Treatment of vemurafenib-resistant brain metastatic melanoma cells with vemurafenib resulted in marginal growth inhibition and apoptosis induction (Fig. 4D and E). Most importantly, combining vemurafenib with the PI3K inhibitor GDC-0941 at equimolar concentrations augmented growth inhibition in these cells (Fig. 4D). Moreover, vemurafenib combined with GDC-0941 induced significant apoptosis in vemurafenib-resistant brain metastatic melanoma cells (Fig. 4E).

## Discussion

In metastatic melanoma, brain metastases occur in the majority of patients and are the most common cause of death. Ongoing clinical studies suggest limited activity of BRAF inhibitors in melanoma brain metastases. We observed in a subset of patients that vemurafenib yielded a partial or complete response in extracerebral metastases, but brain metastases developed. Our immunohistochemical analysis of matched brain and extracerebral metastases demonstrated high AKT activation and loss of PTEN expression in most brain metastases. Astrocyte-conditioned medium stimulated AKT activation and invasiveness in melanoma cells, and inhibition of PI3K-AKT signaling sensitized melanoma cells isolated from a vemurafenib-resistant brain metastasis to vemurafenib. Collectively, these data suggest that brain-derived factors induce activation of the AKT survival pathway and promote the survival and drug resistance of melanoma cells in the brain.

In a series of patients with metastatic melanoma, we observed a difference in the treatment responses of melanoma patients to targeted therapy with vemurafenib: there was partial or complete remission of extracerebral metastases, but development of new cerebral metastases. Many traditional chemotherapeutic agents, as well as newer targeted drugs such as trastuzumab, cannot efficiently cross the blood–brain barrier. The brain is thus regarded as a sanctuary site for metastatic tumor cells, affording them protection from anticancer drugs. Indeed, recent in vitro studies demonstrated that vemurafenib is a substrate for the efflux transporters P-glycoprotein (P-gp) and breast cancer-resistance protein (BCRP) [16]. Furthermore, in vivo studies in mice showed that P-gp and BCRP cooperatively restrict the brain distribution of vemurafenib [16], and that coadministration of the P-gp and BCRP inhibitor elacridar increases the brain penetration of vemurafenib [17]. These experimental data suggest that vemurafenib may reach the brain at subtherapeutic levels. However, magnetic resonance imaging shows uptake of gadolinium contrast dye in melanoma brain metastases and the BRAF inhibitors vemurafenib and dabrafenib show clinical activity, albeit limited, in melanoma patients with brain metastases [10,11], suggesting leakage of the blood–brain barrier in melanoma brain metastases. Altogether, these observations suggest that brain metastases are supported by blood–brain barrier efflux transporters plus factors other than the blood–brain barrier [[18], [19]].

In this study, we observed a patient under treatment with vemurafenib who experienced complete remission of extracerebral metastases while developing a new brain metastasis. This patient benefited when the brain metastasis was excised and vemurafenib treatment was continued. In contrast, a patient who experienced complete remission of extracerebral metastases but developed two small brain metastases which progressed rapidly when vemurafenib was discontinued. These observations support the notion that patients with progressive disease at an isolated site that is addressed by localized therapy may have a survival benefit when vemurafenib is continued [20].

Immunohistochemical analysis of matched brain and extracerebral metastases demonstrated AKT hyperactivation in brain metastases. This observation is in line with a recently published quantitative analysis of AKT activation in melanoma specimens and cell lines using the reverse-phase protein array technology [21]. In the small panel of unmatched distant metastases included in this study, brain metastases were found to have a higher p-AKT expression than distant metastases from the lung and liver. In melanoma, activation of the PI3K-AKT signaling pathway can occur through multiple mechanisms. Various growth factors as well as adhesion molecules may result in activation of this pathway [2,22], AKT, that is the AKT3 isoform, may be overexpressed as a result of copy number increases [23], and the negative regulator of the PI3K-AKT pathway, PTEN, may be inactivated [22]. Indeed, our immunohistochemical analysis showed that metastases with high p-AKT, that is brain metastases, had low PTEN levels. This observation is in line with the above-mentioned study reporting low PTEN and elevated p-AKT levels in brain metastases [21], and suggests that the brain environment suppresses PTEN expression leading to high AKT activation in melanoma cells. Furthermore, BRAF-mutant metastases and cell lines were reported to have higher p-AKT levels than NRAS-mutant metastases and cell lines [21]. However, our mutation analysis revealed no difference in BRAF, NRAS, or KIT mutation status in matched brain and extracerebral metastases of melanoma patients, indicating that hyperactivation of AKT in melanoma brain metastases does not depend on the mutation status of these genes.

In monolayer cultures of brain and extracerebral metastatic cell lines newly derived from melanoma patients, AKT activation and PTEN expression was identical. Furthermore, melanoma cells stimulated by astrocyte-conditioned medium showed higher activation of AKT compared with melanoma cells stimulated by fibroblast-conditioned medium. Astrocyte-conditioned medium increased invasiveness of melanoma cells suggesting that astrocyte-induced AKT activation in melanoma cells promotes invasion of melanoma cells in the brain. However, astrocytes may also contribute to the invasiveness of tumor cells in the brain by producing enzymes such as heparanase that degrade components of the extracellular matrix of the brain [24]. These in vitro data, together with the in vivo observations described above, strengthen the hypothesis that hyperactivation of AKT in melanoma brain metastases is due to the tumor environment. Interestingly, competitive cross-species hybridization of microarray experiments showed that the brain microenvironment induces complete reprogramming of metastasized cancer cells [25]. When xenografted in the brain, all human cancer cell lines tested in this study acquired neuronal expression patterns that can also be induced by culture with astrocytes. When metastatic tumor cells cross the blood–brain barrier, astrocytes are among the first cells to interact with the brain-invading cells. Several experimental studies indicate that astrocytes may contribute to tumor progression in the brain through a variety of different mechanisms, including the secretion of substances that promote tumor cell proliferation and invasion, protection of tumor cells from apoptosis through direct cell–cell interactions, and suppression of adaptive immune responses [18,24]. Specifically, insulin-like growth factor 1 (IGF-1), transforming growth factor beta (TGF-β), and interleukin 6 (IL-6) secreted by astrocytes have been shown to promote proliferation of tumor cells in the brain [26,27]. Thus, astrocyte-derived factors may suppress PTEN expression, activate the AKT survival pathway and promote treatment resistance in melanoma cells in the brain.

Notably, inhibition of PI3K-AKT signaling resensitized melanoma cells isolated from a vemurafenib-resistant brain metastasis to vemurafenib. This observation suggests that the resistance of BRAFV600E-mutated melanoma brain metastases to vemurafenib may be overcome by adding a PI3K inhibitor.

Taken together, our findings suggest that hyperactivation of the AKT survival pathway in melanoma brain metastases is induced by brain-derived factors that promote the survival and drug resistance of melanoma cells in the brain parenchyma. Inhibition of this pathway may be a suitable strategy for enhancing and/or prolonging the antitumor effects of BRAF inhibitors or other anticancer agents in melanoma brain metastases. This hypothesis should prompt experimental studies that analyze the mechanisms of AKT activation in melanoma brain metastases and clinical studies that investigate combinations of PI3K/AKT inhibitors with BRAF/MEK inhibitors or other anticancer agents for treatment of melanoma brain metastases.

## Supporting Information

Table S1. Responses of patients with metastatic melanoma treated with vemurafenib (*n* = 7). **Table S2.** Primer sequences used for mutational analysis of cerebral and matched extracerebral melanoma metastases.

## References

[1] Balch C. M., Gershenwald J. E., Soong S. J., Thompson J. F., Atkins M. B., Byrd D. R. (2009). Final version of 2009 AJCC melanoma staging and classification. J. Clin. Oncol..

[2] Davies H., Bignell G. R., Cox C., Stephens P., Edkins S., Clegg S. (2002). Mutations of the BRAF gene in human cancer. Nature.

[3] Meier F., Schittek B., Busch S., Garbe C., Smalley K., Satyamoorthy K. (2005). The RAS/RAF/MEK/ERK and PI3K/AKT signaling pathways present molecular targets for the effective treatment of advanced melanoma. Front. Biosci..

[4] Chapman P. B., Hauschild A., Robert C., Haanen J. B., Ascierto P., Larkin J. (2011). Improved survival with vemurafenib in melanoma with BRAF V600E mutation. N. Engl. J. Med..

[5] Sosman J. A., Kim K. B., Schuchter L., Gonzalez R., Pavlick A. C., Weber J. S. (2012). Survival in BRAF V600-mutant advanced melanoma treated with vemurafenib. N. Engl. J. Med..

[6] Staudt M., Lasithiotakis K., Leiter U., Meier F., Eigentler T., Bamberg M. (2010). Determinants of survival in patients with brain metastases from cutaneous melanoma. Br. J. Cancer.

[7] Elaimy A. L., Mackay A. R., Lamoreaux W. T., Fairbanks R. K., Demakas J. J., Cooke B. S. (2011). Multimodality treatment of brain metastases: an institutional survival analysis of 275 patients. World J. Surg. Oncol..

[8] Chiarion-Sileni V., Guida M., Ridolfi L., Romanini A., Del Bianco P., Pigozzo J. (2011). Central nervous system failure in melanoma patients: results of a randomised, multicentre phase 3 study of temozolomide- and dacarbazine-based regimens. Br. J. Cancer.

[9] Margolin K., Ernstoff M. S., Hamid O., Lawrence D., McDermott D., Puzanov I. (2012). Ipilimumab in patients with melanoma and brain metastases: an open-label, phase 2 trial. Lancet Oncol..

[10] Dummer R., Rinderknecht J., Goldinger S. M., Wagner I., Mitchell L., Veronese M. L. (2011). An open-label pilot study of vemurafenib in previously treated metastatic melanoma patients with brain metastases. J. Clin. Oncol..

[11] Kirkwood J. M., Long G. V., Trefzer U., Davies M. A., Ascierto P. A., Chapman P. B. (2012). BREAK-MB: a phase II study assessing overall intracranial response rate to dabrafenib (GSK2118436) in patients with BRAFV600E/K mutation-postive melanoma with brain metastases. J. Clin. Oncol..

[12] Lasithiotakis K., Sinnberg T., Schittek B., Flaherty K. T., Kulms D., Maczey E. (2008). Combined inhibition of MAPK and mTOR signalling pathways potently inhibits growth, induces apoptosis and abrogates invasive tumour growth of melanoma cells. J. Invest. Dermatol..

[13] Mancianti M. L., Herlyn M., Weil D., Jambrosic J., Rodeck U., Becker D. (1988). Growth and phenotypic characteristics of human nevus cells in culture. J. Invest. Dermatol..

[14] Meier F., Nesbit M., Hsu M. Y., Martin B., Van Belle P., Elder D. E. (2000). Human melanoma progression in skin reconstructs: biological significance of bFGF. Am. J. Pathol..

[15] Sinnberg T., Lasithiotakis K., Niessner H., Schittek B., Flaherty K. T., Kulms D. (2009). Inhibition of PI3K-AKT-mTOR signalling sensitises melanoma cells to cisplatin and temozolomide. J. Invest. Dermatol..

[16] Mittapalli R. K., Vaidhyanathan S., Sane R., Elmquist W. F. (2012). Impact of P-glycoprotein (ABCB1) and breast cancer resistance protein (ABCG2) on the brain distribution of a novel BRAF inhibitor: vemurafenib (PLX4032). J. Pharmacol. Exp. Ther..

[17] Durmus S., Sparidans R. W., Wagenaar E., Beijnen J. H., Schinkel A. H. (2012). Oral availability and brain penetration of the B-RAF(V600E) inhibitor vemurafenib can be enhanced by the p-glycoprotein (ABCB1) and breast cancer resistance protein (ABCG2) inhibitor elacridar. Mol. Pharm..

[18] Fidler I. J. (2011). The role of the organ microenvironment in brain metastasis. Semin. Cancer Biol..

[19] Gerstner E., Fine R. L. (2007). Increased permeability of the blood-brain barrier to chemotherapy in metastatic brain tumors: establishing a treatment paradigm. J. Clin. Oncol..

[20] Amaravadi R., Kim K., Flaherty K., Chapman P., Puzanov I., Sosman J. (2011). Prolonged response to vemurafenib in patients with BRAFV600E-mutant melanoma with low tumor burden at baseline. Pigment Cell Melanoma Res..

[21] Davies M. A., Stemke-Hale K., Lin E., Tellez C., Deng W., Gopal Y. N. (2009). Integrated molecular and clinical analysis of AKT activation in metastatic melanoma. Clin. Cancer Res..

[22] Madhunapantula S. V., Robertson G. P. (2009). The PTEN-AKT3 signaling cascade as a therapeutic target in melanoma. Pigment Cell Melanoma Res..

[23] Stahl J. M., Sharma A., Cheung M., Zimmerman M., Cheng J. Q., Bosenberg M. W. (2004). Deregulated AKT3 activity promotes development of malignant melanoma. Cancer Res..

[24] Lorger M. (2012). Tumor microenvironment in the brain. Cancers.

[25] Park E. S., Kim S. J., Kim S. W., Yoon S. L., Leem S. H., Kim S. B. (2011). Cross-species hybridization of microarrays for studying tumor transcriptome of brain metastases. Proc. Natl. Acad. Sci. USA.

[26] Seike T., Fujita K., Yamakawa Y., Kido M. A., Takiguchi S., Teramoto N. (2010). Interaction between lung cancer cells and astrocytes via specific inflammatory cytokines in the microenvironment of brain metastasis. Clin. Exp. Metastasis.

[27] Sierra A., Price J. E., Garcia-Ramirez M., Méndez O., López L., Fabra A. (1997). Astrocyte-derived cytokines contribute to the metastatic brain specificity of breast cancer cells. Lab. Invest..

