# Supplementary material for: Targeting hyperactivation of the AKT survival pathway to overcome therapy resistance of melanoma brain metastases
Source: Cancer Med. 2013 Feb 3;2(1):76–85. doi: 10.1002/cam4.50 (PMC3797558; doi:10.1002/cam4.50)
Supplement: Table S1. — Responses of patients with metastatic melanoma treated with vemurafenib (n = 7). Table S2. Primer sequences used for mutational analysis of cerebral and matched extracerebral melanoma metastases. [file cam0002-0076-SD1.doc]

**Supplementary Table1: Responses of Patients with Metastatic Melanoma Treated with Vemurafenib (n=7)**

| Patient | Metastases | Vemurafenib | Response |
| --- | --- | --- | --- |
| 1 | 08/2010  Lung, lymph nodes, mediastinum | 9 months | 05/2011  Extracerebral complete response.  Cerebral progression |
| 2 | 06/2010  Lung, liver, lymph nodes | 14 months | 08/2011  Extracerebral complete response.  Cerebral progression |
| 3 | 08/2010  Subcutis, lung | 3 months | 11/2010  Extracerebral complete response.  Cerebral progression |
| 4 | 08/2011  Mediastinum, lymph nodes | 3 months | 11/2011  Extracerebral mixed response with regression in the mediastinum and lymph nodes.  Cerebral progression |
| 5 | 07/2011  Liver, pancreas, lymph nodes | 2 months | 09/2011  Extracerebral mixed response with regression in the liver, pancreas, and lymph nodes.  Cerebral progression |
| 6 | 03/2011  Spleen, lung, adrenal gland, muscles | 2 months | 05/2011 Extracerebral mixed response with regression in the spleen.  Cerebral progression |
| 7 | 11/2011  Liver, lung, bones | 2 months | 01/2012  Extracerebral mixed response with regression in the liver and lung.  Cerebral progression |

**Supplementary Table 2:** Primer sequences used for mutational analysis of cerebral and matched extracerebral melanoma metastases

| Primer | Sequence |
| --- | --- |
| BRAF-ex-15-R | CCAAAAATTTAATCAGTGGA |
| BRAF-ex-15-F | TCATAATGCTTGCTCTGATAGGA |
| KIT-ex-11-R | AAACAAAGGAAGCCACTGGA |
| KIT-ex-11-F | TGTTCTCTCTCCAGAGTGCTCTAA |
| KIT-ex-13-R | AGCAAGAGAGAACAACAGTCTGG |
| KIT-ex-13-F | TCATCAGTTTGCCAGTTGTGC |
| KIT-ex-17-R | TCGAAAGTTGAAACTAAAAATCC |
| KIT-ex-17-F | TCATTCAAGGCGTACTTTTG |
| KIT-ex-18-R | CAAGGAAGCAGGACACCAAT |
| KIT-ex-18-F | TCATTTCAGCAACAGCAGCAT |
| NRAS-Cod61-R | TGACTTGCTATTATTGATGG |
| NRAS-Cod61-F | TGGTGAAACCTGTTTGTTGGA |
